# Supplementary material for: Effects of Light and Oxygen on Chlorophyll d Biosynthesis in a Marine Cyanobacterium Acaryochloris marina
Source: Plants (Basel). 2022 Mar 29;11(7):915. doi: 10.3390/plants11070915 (PMC9003380; doi:10.3390/plants11070915)
Supplement: Supplementary file 1 [file plants-11-00915-s001.zip › plants-1606905-supplementary.pdf]

Effects of light and oxygen on chlorophyll *d* biosynthesis in a marine cyanobacterium *Acaryochloris marina*

Yuki Tsuzuki [1], Yusuke Tsukatani [2], Hisanori Yamakawa [1], Shigeru Itoh [3], Yuichi Fujita [1], and Haruki Yamamoto [1,*]

[1] Graduate School of Bioagricultural Sciences, Nagoya University; duffko0614@gmail.com (Yuki T.); yamakawa008@gmail.com (Hisanori Y.); fujita@agr.nagoya-u.ac.jp (Y. F.)
[2] Japan Agency for Marine-Earth Science and Technology (JAMSTEC); tsukatani@jamstec.go.jp (Yusuke T.)
[3] Graduate School of Science, Nagoya University; itoh@bio.phys.nagoya-u.ac.jp (S. I.)
* Correspondence: haruki@agr.nagoya-u.ac.jp; Tel.: +81-52-789-4090

Supplemental information

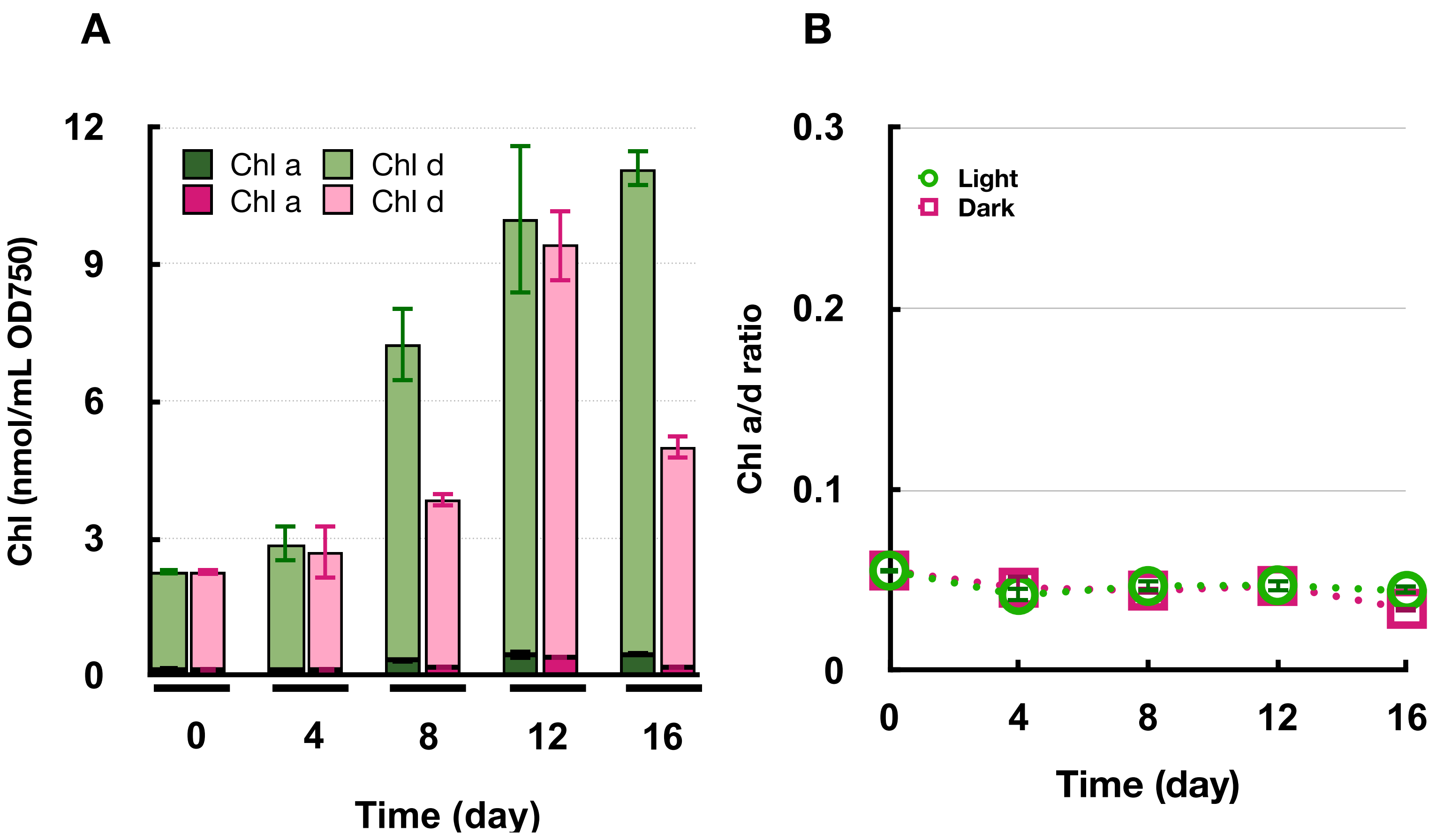

Figure S1. Chl *a/d* content in the dark-grown cultures of *A.* sp. MBIC10699
(A) The amounts of Chl *a* (dark green/dark pink) and Chl *d* (green/pink) were measured on days 0, 4, 8, 12, and 16 of *A.* sp MBIC10699 cells in photo-autotrophic (green) and dark-heterotrophic (pink) conditions, respectively. (B) The ratio of cellular Chl *a*/*d* amount in photo-autotrophic conditions (green circle) and dark-heterotrophic conditions (pink square).

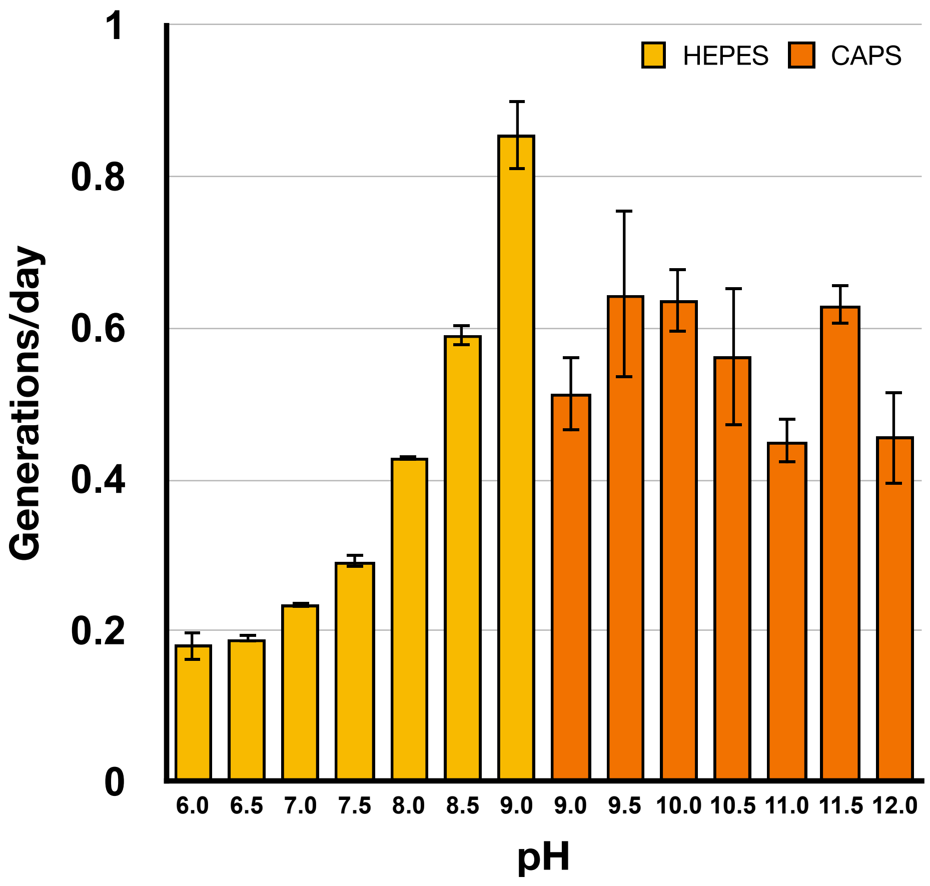

Figure S2. Compalison of growth in *A. marina* under various pH conditions

A. marina was cultured under various pH conditions, and the number of generations/day was calculated from the growth rate of the exponential phase. To adjust pH in IMK medium, HEPES (yellow) and CAPS (orange) buffer were used.
